# Improvements to the Red List Index

**DOI:** 10.1371/journal.pone.0000140

**Published:** 2007-01-03

**Authors:** Stuart H.M. Butchart, H. Resit Akçakaya, Janice Chanson, Jonathan E.M. Baillie, Ben Collen, Suhel Quader, Will R. Turner, Rajan Amin, Simon N. Stuart, Craig Hilton-Taylor

**Affiliations:** 1 BirdLife International, Cambridge, United Kingdom; 2 Applied Biomathematics, Setauket, New York, United States of America; 3 Conservation International/Center for Applied Biodiversity Science-World Conservation Union (IUCN)/Species Survival Commission Biodiversity Assessment Unit, IUCN Species Programme, Center for Applied Biodiversity Science, Conservation International, Washington, D. C., United States of America; 4 Institute of Zoology, Zoological Society of London, London, United Kingdom; 5 Department of Zoology, University of Cambridge, Cambridge, United Kingdom; 6 Center for Applied Biodiversity Science, Conservation International, Washington, D. C., United States of America; 7 World Conservation Union (IUCN) Species Programme, Cambridge, United Kingdom; 8 Royal Society for the Protection of Birds, Sandy, United Kingdom; Dalhousie University, Canada

## Abstract

The Red List Index uses information from the IUCN Red List to track trends in the projected overall extinction risk of sets of species. It has been widely recognised as an important component of the suite of indicators needed to measure progress towards the international target of significantly reducing the rate of biodiversity loss by 2010. However, further application of the RLI (to non-avian taxa in particular) has revealed some shortcomings in the original formula and approach: It performs inappropriately when a value of zero is reached; RLI values are affected by the frequency of assessments; and newly evaluated species may introduce bias. Here we propose a revision to the formula, and recommend how it should be applied in order to overcome these shortcomings. Two additional advantages of the revisions are that assessment errors are not propagated through time, and the overall level extinction risk can be determined as well as trends in this over time.

## Introduction

In response to the accelerating rate of biodiversity loss, and the far-reaching impacts of this, the governments of 190 countries have pledged to significantly reduce the rate of biodiversity loss by 2010 [1]. This has led to increasing requirements for indicators that can chart the rate of biodiversity loss [2], [3]. In response, the World Conservation Union (IUCN) and its partner organisations developed an indicator - the Red List Index (RLI; [4]) - based on the IUCN Red List of Threatened Species™.

The IUCN Red List is widely recognised as the most authoritative and objective system currently available for classifying species in terms of their risk of global extinction [5]–[9]. It uses quantitative criteria based on population size, rate of decline, and area of distribution to assign species to categories of relative extinction risk [10]. These criteria are clear and comprehensive, yet are sufficiently flexible to deal with uncertainty [11]. Assessments of individual species using these criteria must be supported by a wealth of documentation, including information on range, occurrence, population, trends, habitat preferences, threats, conservation actions in place and needed [8]. The Red List is also becoming increasingly comprehensive, with all species now assessed in several major classes (birds, mammals, amphibians, conifers and cycads) and global assessments underway for all reptiles, marine species in several groups (including sharks and coral-reef fish), several freshwater groups, and selected plant groups (initially, legumes and trees).

The RLI uses information from the IUCN Red List to measure the projected overall extinction risk of sets of species and to track changes in this risk [4], [12], [13]. It is based on the proportion of species in each category on the Red List, and changes in this proportion over time resulting from genuine improvement or deterioration in the status of individual species. The RLI was initially designed and tested using data on all bird species from 1988–2004 [4], and has since been applied to amphibians [13], with a global mammal RLI in preparation. By 2010, RLI trends will also be available for all conifers and cycads, and for a more representative set of taxa based on a random sample of all vertebrates and selected plant groups. Baseline estimates for reptiles and selected freshwater fish, plant and marine groups will also be available. As well as tracking global trends, the RLI can be disaggregated to show trends for species in different biogeographic realms, political units, ecosystems, habitats, taxonomic groups and for species relevant to different international agreements and treaties.

The RLI has been widely recognised as an important component of the suite of indicators needed to track progress towards the 2010 target [3], [8], [14]–[17]. Consequently, an indicator on ‘trends in the status of threatened species’ has been moved into the top group of indicators for ‘immediate testing’ by the Convention of Biological Diversity (CBD) Subsidiary Body on Scientific, Technical and Technological Advice (SBSTTA; [16]). In addition, RLIs based on the relevant sets of species are currently being considered for adoption by the Ramsar Convention on Wetlands, the Convention on Migratory Species (CMS), the Agreement on the Conservation of Albatrosses and Petrels under the CMS, and for European threatened species through the Streamlining European Biodiversity Indicators-2010 initiative, which is coordinated by the European Environment Agency, the European Centre for Nature Conservation and UNEP-WCMC (the World Conservation Monitoring Centre).

Given this increasing recognition and usage, it is important that the RLI performs well as an indicator, for example, by meeting the criteria for successful indicators described by Gregory et al. [18]. Further application of the RLI (and in particular, consideration of its application to non-avian taxa) has, however, revealed some shortcomings in the original formula and approach. We describe these here and recommend revisions that address them to improve the RLI formulation.

## Analysis

### The original RLI formulation and its shortcomings

The original RLI formula was defined as follows: 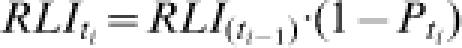


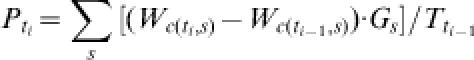


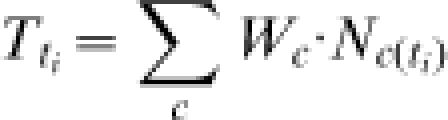
where *RLI_ti_* is the value of the RLI at time *t_i_*; *P*(*t_i_*) is the proportional genuine change in threat status at time *t_i_*; *W_c_* is the weight for category *c* (weights increase with threat); *c*(*t_i_, s*) is the threat category of species *s* at time *t_i_*; *G_s_* = 1 if change (from *t*_(*i*−1)_ to *t_i_*) in category of species *s* is genuine (otherwise *G_s_* = 0); T(*t_i_*) is total threat score at time *t_i_*, where *t_i_* is the year of the *i*th assessment (assessments are not necessarily made every year); *N_c_* (*t_i_*) is the number of species in threat category *c* at time *t_i_*; and *RLI_ti−1_* = 100 for the first year of assessment. Larger values of *RLI_ti_* indicate a better overall conservation status for the set of species.

Although this original RLI formulation meets many of the needs for an indicator of biodiversity loss, further testing and application under different conditions have revealed three shortcomings of this approach and its recommended application:

#### 1. The RLI performs inappropriately once it has reached zero

**Figure 1:**
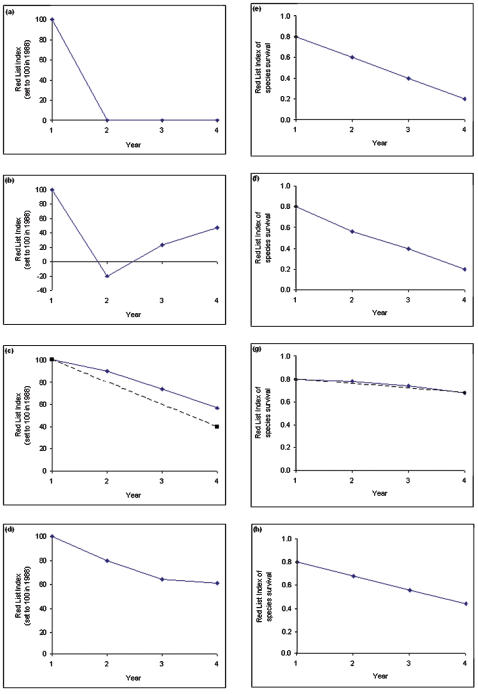
RLIs using the original formulation (left-hand graphs) and the revised formulation (right-hand graphs) for three hypothetical examples: (A and E) a set of five species, which are all classified as Near Threatened in year 1, Vulnerable in year 2, Endangered in year 3, and Critically Endangered in year 4; (B and F) the same scenario, except that one species jumps from Near Threatened to Vulnerable by year 2; (C and G) a set of ten Near Threatened species in year 1, with one species moving into Vulnerable in each subsequent year, and then up through the categories one step at a time each year thereafter; the dotted line shows the RLI as would be calculated if the same set of species were assessed for the IUCN Red List in years 1 and 4 only; (D and H) ten Near Threatened species that deteriorate by 0.1 category per species per year (i.e. one species per year moves up a category), plus ten Near Threatened species that deteriorate at ten times this rate (i.e. all ten species per year move up a category) and that are not assessed until year 3.

The original formula was developed and tested using data on birds from 1988–2004. Over these 16 years, this set of species showed an important, but relatively modest, proportional deterioration in status (as measured through Red List categories), and the RLI value declined by 6.9% over the period [4]. However, we now recognise that problems may arise when a set of species undergoes a large proportional deterioration in status. If the RLI value for a set of species declines by 100% (to exactly zero), it cannot subsequently change. This can happen if *P_ti_* has a value of 1, which occurs when the average threat score is double that of the previous assessment (Eq. 2). Under these circumstances, *RLI_ti_* becomes 0, and cannot subsequently change (Eq. 1). Figure 1A illustrates this for a hypothetical set of five species, which are all classified as Near Threatened in year 1, Vulnerable in year 2, Endangered in year 3, and Critically Endangered in year 4. The RLI value declines by 100% between years 1 and 2, but it subsequently remains at zero despite continuing deterioration in the set of species. Worse, if the RLI value decreases below zero, then further deterioration in the status of the set of species causes an increase in the RLI value instead of a decrease as would be expected. This can happen if *P_ti_* has a value greater than 1, which occurs when the average threat score is more than double that of the previous assessment (Eq. 2). Then, *RLI_ti_* becomes negative (Eq 1); any subsequent deterioration (*P_ti_*>0) leads to an increase in the index value rather than a decrease, and any improvement (*P_ti_*<0) leads to a decrease in the index value rather than an increase. Figure 1B illustrates this for the same hypothetical set of five species, which deteriorate in status at the same rate as in the previous example, except that one species jumps from Near Threatened to Vulnerable by year 2. In this case, the RLI value shows a positive trend after year 2, despite the fact that the status of all the species continues to worsen.

This situation is not merely hypothetical. A preliminary RLI for the world's amphibians for 1980–2004 showed a decline of 104.6% (when weighting categories by relative extinction risk, i.e. Least Concern = 0, Near Threatened = 0.0005, Vulnerable = 0.005, Endangered = 0.05, Critically Endangered = 0.5, Extinct and Extinct in the Wild = 1; see [4] for further details). This problem would be more likely to occur when calculating RLIs over longer time periods, for groups deteriorating at a faster rate, and for groups with fewer species (where the rapid deterioration of a small number of species can lead to the average threat score becoming more than double that of the previous assessment).

#### 2. RLI values are affected by the frequency of assessments

Under the original formulation, the RLI value at a particular time point is dependent on the number of assessments since the baseline year. In other words, the frequency of assessments influences RLI values. This is because the RLI value is calculated in relation to the value for the previous assessment. Figure 1C shows a hypothetical example for ten Near Threatened species in year 1. In each subsequent year, one species moves into Vulnerable, and then continues to move up one category per year. By year 4 there is one Critically Endangered species, one Endangered, one Vulnerable and seven Near Threatened species. By comparison, the dotted line in Figure. 1c shows the situation if assessments had been carried out in years 1 and 4 only. With the same set of species having undergone the same status changes, a substantially different RLI value results. This presents great difficulties if RLIs are compared for two or more sets of species that are assessed with different frequencies. This is a highly likely scenario as it is difficult to synchronise major initiatives (such as the Global Mammal Assessment and Global Amphibian Assessment), involving thousands of scientists and running on time-cycles determined by logistics and funding opportunities.

#### 3. Newly evaluated species may introduce bias

Any approach to calculating an RLI has to handle situations in which species being evaluated for the first time are added to the original set of species that were used to calculate the index. Species may be added because: (a) they are newly recognised taxonomically; or (b) they were previously assessed as Data Deficient. Under the original approach, such species contribute to the index value only when they are assessed for the second time, and only from that point onwards. Hence, if the extinction risk of a suite of newly added species is changing at a different average rate from the original set, they will contribute to a ‘false’ change in the RLI trend (i.e. one that did not reflect the status changes of the overall set of species). Figure 1D illustrates this with a hypothetical example starting with ten Near Threatened species which deteriorate by 0.1 category per species per year (i.e. one species per year moves to the next highest category of extinction risk). Another set of ten species are deteriorating at ten times this rate (i.e. all ten species per year move to the next highest category of extinction risk), but this second set is not assessed until year 3 (by which time all the species are Endangered). An RLI using the original approach shows a sharp reduction in the rate of decline after year 3 (Figure 1D), rather than the expected increase in the rate of decline (assuming no back-casting: see below).

This problem is likely to be common in practice because it is quite possible that newly evaluated species will differ in the average rate they are slipping towards extinction. They certainly often differ in their average extinction risk compared to the overall species set. Newly split or newly described species tend to have smaller ranges than their congeners (and hence are more likely to be threatened), while Data Deficient species are often concentrated in parts of the world suffering severe environmental threats but where little information is available (e.g. Somalia and New Guinea for birds; BirdLife International unpublished data). For birds, 45 Data Deficient species have been re-evaluated since 1994. Excluding seven that are no longer recognised taxonomically, 84% were assessed as Near Threatened or threatened (BirdLife International unpublished data), compared to 12.3% for extant birds as a whole [19].

### A revised RLI formulation

In response to these shortcomings, and to suggestions for how to make the RLI easier to interpret, we here propose a revision to the original formula.

We define the revised RLI as:
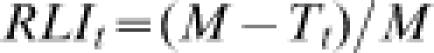
where *M* is the ‘maximum threat score’, i.e. the number of species multiplied by the maximum category weight (*W*_EX_, which is the weight assigned to extinct species; this equals 5 using the recommended ‘equal steps’ weights, with Critically Endangered = 4, Endangered = 3, Vulnerable = 2, Near threatened = 1, Least Concern = 0; see [4] for further discussion). Thus,
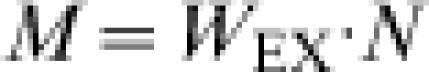
where *N* is the total number of assessed species, excluding those considered Data Deficient and those assessed as Extinct in the year the set of species was first assessed. (Alternatively, if RLIs for different sets of species are being compared, species that have gone extinct prior to the earliest year of assessment for any group would be excluded.)

The ‘current threat score’ (*T*) is defined as:
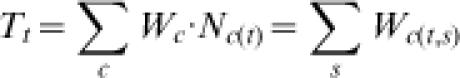
The alternative formulations in equation 6 give the same result; the first is a summation over all categories, from Least Concern to Extinct, and the second is a summation over all assessed non-Data Deficient species. Thus, the maximum possible value of *T* is *M*, and RLI values can vary from 0 (all species are Extinct) to 1 (all species Least Concern).

Equations 4–6 can be combined into a single equation as follows:
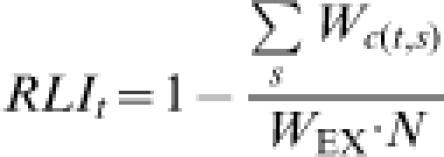
Application of this formulation requires that (a) exactly the same set of species is included in all time steps, and (b) the only category changes are those resulting from genuine improvement or deterioration in status (i.e. excluding changes resulting from improved knowledge or taxonomic revisions; see [4], [13]for further details). In many cases, species lists will change slightly from one assessment to the next (e.g. owing to taxonomic revisions). We therefore recommend that this formulation be applied in conjunction with a new approach, which we term ‘back-casting’, of retrospectively adjusting earlier Red List categorisations using current information and taxonomy. This allows the preconditions to be met by assuming that the current Red List categories for the taxa have applied since the set of species was first assessed, unless there is information to the contrary that genuine status changes have occurred. Such information is often contextual, e.g. relating to the known history of habitat loss within the range of the species (see below for further discussion).

Occasionally, there is insufficient information available to back-cast categories of extinction risk for a newly added species (i.e. a species for which we lack confidence that genuine status changes would be detected). Such a species would not be added until it was assessed subsequently for a second time, at which point earlier assessments may be back-cast by extrapolating recent trends in population, range, habitat and threats, supported by additional information.

## Discussion

### Strengths of the revised formulation

Application of the revised formulation and approach solves all three problems outlined above, as shown in Figure 1. RLI values cannot become fixed to zero (Figure 1E; see below for discussion of the particular meaning of zero under the new formulation) or become negative (Figure 1F); they are not affected by the frequency of assessments (Figure 1G), and species evaluated for the first time that differ in average extinction risk or in the rate of change of extinction risk do not introduce spurious trends (Figure 1H). In addition, it has two further advantages:

#### 1. Assessment errors are not propagated

Applying the new formula as described above means that the RLI value at a particular time reflects the best understanding of the overall extinction risk of the set of species, and a series of RLI values reflect the degree to which this risk has changed over time. By contrast, under the original approach, RLI values also reflected historical errors in extinction risk estimates. Both the original and new approach assume that all (or a substantial proportion of) genuine status changes that are large enough for a species to cross the thresholds for a new Red List category will be detected. Both approaches also allow such genuine status changes to be identified after a delay, and retrospectively incorporated into the index. However, for species that haven't undergone genuine change, the new formulation additionally allows assessment errors resulting from incomplete or inaccurate knowledge to be corrected, by assuming that the most recent (and best-informed) evaluations have applied since the first assessment unless genuine status changes have been detected. By contrast, the original formulation takes as its starting point the categories assigned when the set of species was first assessed, including those that were incorrect owing to inaccurate or incomplete knowledge. Hence the original approach produces an RLI whose trends also reflect errors and inaccuracies in earlier knowledge. The degree to which this produces bias will increase with time since the first assessment.

**Figure 2:**
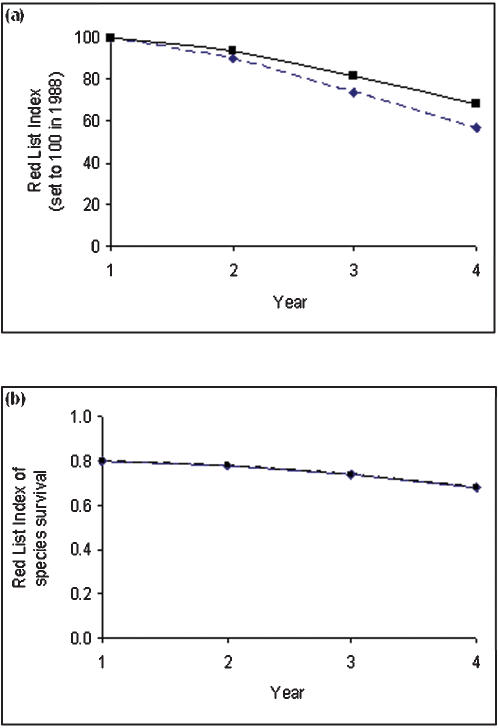
RLI using (A) the original formulation; and (B) the new formulation for the same hypothetical set of 10 species as in Figure 1c and g. The dotted line represents the RLI that would be calculated if five Near Threatened species had been misclassified as Vulnerable in year 1 owing to poor knowledge, and if this error was not corrected until year 4. The original formulation produces an RLI that is substantially lower in value than reality because it propagates errors resulting from incomplete knowledge in earlier assessments. The new formulation does not suffer from this effect.

**Figure 3:**
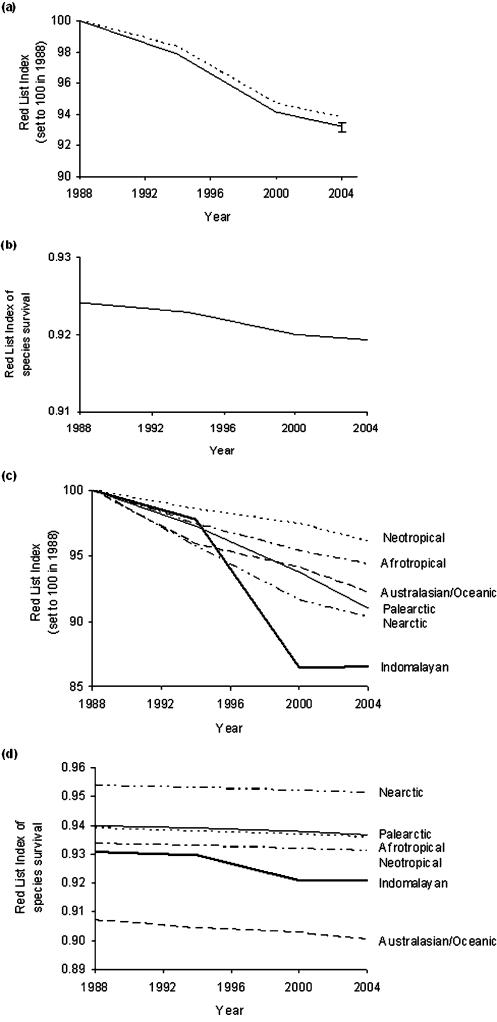
RLIs using the original formulation (left-hand graphs) and the revised formulation (right-hand graphs) for (A–B) the world's birds (n = 9,824 non-Data Deficient species: 99.2% of all extant species) and (C–D) birds in different biogeographic realms. Under the revised formula, an RLI value of 1.0 equates to all species being categorised as Least Concern, and hence that none are expected to go extinct in the near future; an RLI value of zero indicates that all species have gone Extinct. In Figure 3A, the dotted line represents the RLI using the same original formula, but incorporating back-casting using the latest and best-informed evaluations. It results in a substantially higher value by 2004 because the original approach propagates errors resulting from incomplete knowledge in earlier assessments. By 2004, the difference is comparable to the size of the error bars calculated from estimates of the magnitude of the error introduced by delays in knowledge becoming available to assessors [13].

Figure 2 illustrates this with the same hypothetical example as used in Figures 1C and 1G. If this is assumed to represent ‘reality’, it can be compared to an RLI that would result if we had misclassified five Near Threatened species as Vulnerable in year 1 owing to poor knowledge, and if this error was not corrected until year 4. The original formulation produces an RLI that is substantially lower than reality, whereas the revised formulation does not suffer this effect. A real example is shown in Figure 3A, where two versions of the RLI for the world's birds for 1988–2004 is shown, with (dotted line) and without (solid line) incorporating back-casting. As time passes, the divergence between the two lines, and hence the degree of bias, increases. For birds, the scale of this bias is comparable to the magnitude of the error introduced by delays in knowledge becoming available to assessors (see below): the error bars calculated based on estimates of the magnitude of this phenomenon (as shown in Figure 3; see [13] Figure 1) are of a comparable size. If presenting an RLI for a single set of species, this phenomenon is not too problematic. However, it would be an important source of bias when comparing two sets of species that differ in the accuracy of knowledge about their status.

#### 2. Overall extinction risk and rate of change can be distinguished

The revised RLI is scaled such that a value of 1 indicates that all species are Least Concern, and an RLI value of 0 indicates that all species have gone extinct. An intermediate value indicates how far the set of species has moved overall towards extinction. Thus the revised RLI allows comparisons between sets of species in both their overall *level* of extinction risk (i.e. how threatened they are on average), and in the *rate* at which this changes over time. This represents an advantage over the original formula, in which RLIs for different sets of species are all set to 100 in the baseline year, masking any overall differences in extinction risk. Figure 3 shows the revised RLI using the original and revised formulas for all birds for 1988–2004, and for birds in different biogeographic realms during the same period. The latter figures highlight the difference between the formulas when comparing multiple sets of species. Under the original formula, birds in the Nearctic and Indomalayan realms appeared to have undergone the largest proportional deterioration in status. The RLI using the revised formula also highlights the plight of Indomalayan species, but shows that Nearctic species are the least threatened on average, and that those in the Australasian/Oceanic realms are also of particular concern.

### A weakness of the revised formulation?

The new formulation adopts the principle of back-casting extinction risk categories for species to earlier assessment dates using the most up-to-date and best-informed evaluations. Conceivably, this could introduce bias for newly evaluated species if it was more difficult to detect genuine changes for such species since the date the RLI was first calculated for the complete set of species. In other words, one could add a suite of newly evaluated Data Deficient or taxonomically split species, assign their currently evaluated categories to previous assessment dates, and fail to detect that some had undergone genuine status changes since the date the RLI was first calculated. We believe that such a scenario will arise infrequently in completely assessed groups, based on consideration of the 1,961 birds, mammals and amphibians currently assessed as Data Deficient, plus those bird species that have been newly assessed owing to taxonomic revisions since the first global assessment of birds in 1988.

For a Data Deficient species to be reassigned to a different category requires that information is available on its current status, usually including its range, population size, trends, habitat preferences and threats. This usually also necessitates understanding the recent historical status of the species. Inferences about past trends are often based on contextual information such as analysis of satellite imagery to evaluate the extent and timing of habitat loss within the range of the species. The majority of Data Deficient species are so-classified because there is little or no recent information on their status, owing to a lack of recent surveys. Once these are completed, it is usually straightforward to assess how their status may have recently changed. Data Deficient species are often concentrated in poorly known parts of the world. For example, 10% of Data Deficient bird species are restricted to Somalia (and in some cases adjacent parts of Ethiopia). Owing to the security situation, there has been no information on the status of these species for two decades. Once peace returns, it will be possible to reassess them based on up-to-date surveys of their range and population, combined with data on habitat loss, and at the same time to determine whether the status of any of them may have changed sufficiently since 1988 to have crossed the thresholds for a different Red List category.

A specific example is provided by Long-legged Thicketbird *Trichocichla rufa*. This species is endemic to Fiji, where it had been known from four old specimens, a handful of unconfirmed sightings and one specimen from 1974. It had been considered too poorly known to evaluate against the Red List categories and criteria. However, it was rediscovered in 2002, and surveys in the following years found it to be locally common at several sites, but patchily distributed [20]. It was consequently reassessed as Endangered in 2006 owing to its small population (estimated to number 50–250 individuals), triggering criterion D1 [21]. The population was considered to be stable, and there is no reason to suggest that it has changed significantly in recent years. Therefore, for the purposes of calculating the RLI, the category of Endangered was back-cast to the 1988 assessment with a high degree of confidence.

As noted above, in cases where it is felt there is insufficient information to back-cast categories for earlier assessments, species can be excluded until they are assessed for a second time, at which point earlier assessments may be back-cast with greater confidence. We consider that the inaccuracies and biases produced by the approach underlying the original formula (i.e. those resulting from propagation of previous assessment errors and the incorporation of newly evaluated species) to be substantially greater than those introduced by the principle of back-casting used by the new formula, although it is not possible to test this explicitly until we have longer time series of data from a range of taxonomic groups.

### Sources of uncertainty in RLIs

We recognise four main types of uncertainty in RLI values and trends: deriving from (a) inadequate, incomplete or inaccurate knowledge; (b) delays in knowledge becoming available to assessors; (c) inconsistency between assessors; and (d) Data Deficient species. (A fifth source applies only to RLIs based on sampled sets of species, an approach that is still being developed to increase taxonomic breadth of RLIs, and which will be discussed elsewhere).

(a) RLI values may be incorrect because of errors in the Red List categories assigned to species owing to poor knowledge. However, this potential problem is minimised by two aspects of the Red Listing process. Firstly, IUCN Red List categories are relatively coarse measures of extinction risk, with large differences in the quantitative thresholds under each criterion. For example, an estimate that a species' range encompasses 500 km^2^ may well be uncertain to some degree, but the true value could be as small as 100 km^2^ or as large as 4,999 km^2^ and the species would still be accurately classified as Endangered under criterion B (assuming the other qualifiers were also met). Secondly, Red List assessments are only carried out every four years or more (4–6 years for the bird data used in Figure. 3), so the timing of status changes needs to be accurate only to within this timeframe. For example, there may be uncertainty around the estimate that a particular species' population fell below 1,000 individuals (and hence qualified as Vulnerable under criterion D1) in 1990, but the true date could fall anywhere in the period 1988–1994 (when the first and second complete assessments for birds were carried out) and the status change would still be correctly assigned to the appropriate time-period. Hence, because estimates of extinction risk are assigned to classes that are broad in magnitude and timing, uncertainty resulting from inadequate knowledge is considerably reduced.

(b) Red List classifications (and hence RLI values) may be incorrect because accurate knowledge of the species has not yet reached the evaluators. However, the revised formula allows such delayed knowledge to be reflected in the RLI (through back-casting) so that it represents the best-informed understanding of the status of the set of species and how this has changed over time. Furthermore, such delays apply to a small proportion of status changes (e.g. 13% of those for the 1994–2000 period for birds), and this proportion is decreasing for birds at least [4]. This trend is likely to continue owing to an expanding network of scientists across the world providing detailed and up-to-date information for an increasing number of species to the IUCN Red List, and with improving and faster channels of communication (e.g. BirdLife's web-based Globally Threatened Bird discussion forums: www.birdlifeforums.org).

(c) Inconsistent application of the Red List categories and criteria between assessors could introduce bias and uncertainty into RLIs (see, e.g. [7], [22]. However, assessments are now required to have supporting documentation detailing the best available data, with justifications, sources, and estimates of uncertainty and data quality [23]. Red List Authorities are appointed to organise independent scientific review of the assessments and to ensure consistent categorisation between species, groups, and assessments. For many classes of organisms, all assessments are now coordinated through small centralised teams (e.g. as part of the Global Amphibian Assessment and Global Mammal Assessment, or through BirdLife International) to ensure standardisation and consistency in the interpretation of information and application of the criteria. Furthermore, a user's working group and the IUCN Red List Programme Office work to ensure consistency between the major taxonomic groups. Finally, a Red List Standards and Petitions Subcommittee monitors the process and resolves challenges and disputes to listings.

(d) Species that are too poorly known for the Red List criteria to be applied to are assigned to the Data Deficient category, and excluded from the calculation of the RLI. For birds, only 0.8% of extant species are evaluated as Data Deficient (see above), compared with 24% of amphibians [19]. If Data Deficient species comprise a substantial proportion of the total set and if these species differ in the rate at which their extinction risk is changing, the RLI may give a biased picture of the changing extinction risk of the overall set of species. The degree of uncertainty this introduces cannot be quantified until a significant proportion of Data Deficient species have been re-assigned to other Red List categories and then reassessed. It is recommended that the proportion of species that are assessed as non-Data Deficient should be stated alongside all RLI graphs.

Techniques are already available to calculate confidence limits based on the uncertainty associated with delays in knowledge acquisition [4]. We consider that inadequate knowledge is likely to be the most important source of uncertainty in most taxonomic groups. We propose to determine its magnitude, and hence to calculate confidence limits, for each RLI by using established techniques for incorporating uncertainty into Red List assessments, i.e. using the RAMAS® software to evaluate the range of possible Red List categories for a sample of species for each assessment [24].

### Interpretation of the RLI

The RLI measures the rate of biodiversity loss, rather than the state of biodiversity. Although some of the Red List criteria are based on absolute population size or range size, others are based on rates of decline in these values or combinations of absolute size and rates of decline. These criteria are used to assign species to Red List categories that can be ranked according to relative projected extinction risk, and the RLI is calculated from changes between these categories. Hence an RLI value is an index of *the proportion of species expected to remain extant in the near future in the absence of any conservation action* (using ‘equal steps’ weights; the RLI value will match this proportion using the ‘extinction risk’ weights; see [4] for further details). The ‘near future’ cannot be quantified exactly, because it depends on the generation times (as defined by [10]) of each of the species contributing to the index, but it most cases the period can be taken to be in the range of 10–50 years.

A downward trend in the RLI over time means that the expected rate of future species extinctions is worsening (i.e. the rate of biodiversity loss is increasing). An upward trend means that the expected rate of species extinctions is abating (i.e. the rate of biodiversity loss is decreasing), and a horizontal line means that the expected rate of species extinctions is remaining the same, although in both cases it does not mean that biodiversity loss has stopped. Hence, to show that the global target of significantly reducing the rate of biodiversity loss by 2010 [1] may have been met, an upward RLI trend is needed at the very least. To show that the European target of halting biodiversity loss by 2010 [25] may have been met, the RLI value must reach 1.0 (assuming that speciation rates are too slow to be relevant in this context, and excluding the small number of species classified as Vulnerable under criterion D2 for which the potential threat is not anthropogenic).

As with other biodiversity indicators, the RLI captures trends in one particular aspect of biodiversity, although for the RLI it is one with a great deal of resonance with the public and decision-makers: the rate that species are moving towards extinction and becoming extinct. The RLI does not capture particularly well the deteriorating status of common species that are declining slowly as a result of general environmental degradation. Indicators based on population trends are better suited for this, and show finer temporal resolution (e.g. [18], [26]). To measure progress towards the 2010 target, a suite of complementary indicators will be required [14]. The RLI forms an important component of this suite, and will be made considerably more robust and more widely applicable by the revisions we have proposed here.
